# Cancer risk in hospitalised psoriasis patients: a follow-up study in Sweden

**DOI:** 10.1038/sj.bjc.6605027

**Published:** 2009-04-07

**Authors:** J Ji, X Shu, K Sundquist, J Sundquist, K Hemminki

**Affiliations:** 1 Center for Primary Health Care Research, Lund University, Sweden; 2 Center for Family and Community Medicine, Karolinska Institute, Huddinge, Sweden; 3 Division of Molecular Genetic Epidemiology, German Cancer Research Center (DKFZ), Heidelberg, Germany

**Keywords:** psoriasis, national databases

## Abstract

We examined overall and specific cancer risks among Swedish subjects who had been hospitalised one or more times for psoriasis. A database was created by identifying such patients from the Swedish Hospital Discharge Register and linking them with the Cancer Registry. Follow-up of patients was carried out from the last hospitalisation through 2004. A total of 15 858 patients were hospitalised for psoriasis during 1965–2004, of whom 1408 developed cancer, giving an overall standardised incidence ratios (SIRs) of 1.33. A significant excess was noted for squamous cell skin cancer, and for cancers of the upper aerodigestive tract, oesophagus, stomach, liver, pancreas, lung, kidney and bladder as well as non-Hodgkin lymphoma. Many of these may reflect the effects of alcohol drinking and tobacco smoking. Patients with multiple hospitalisations showed high risk, particularly for oesophageal (SIR 6.97) and skin (SIR 4.76) cancers.

Psoriasis is a chronic disorder that affects the skin and joints variying from minor localised patches to complete body coverage (Ritchlin, 2007). Its prevalence in Western populations is estimated to be around 2–3% (Gudjonsson and Elder, 2007). The cause of psoriasis is poorly understood, but is believed to include a genetic component (Brandrup *et al*, 1982; Duffy *et al*, 1993; Krueger and Ellis, 2005); concordance in monozygotic twins is about 70%, whereas in dizygotic twins this is only 20% (Krueger and Ellis, 2005). Psoriasis is an immune-mediated disorder, for which T cells are abnormally activated to release cytokines, which cause inflammation and rapid production of skin cells (Griffiths and Voorhees, 1996). It is not clear what factors trigger the activation of T cells. Stress, excessive alcohol consumption and smoking can aggravate psoriasis (Kimball *et al*, 2005). The choice among many potential treatment options depends upon the type of psoriasis, its location and severity (Boehncke *et al*, 2006). Normally, medications with the least adverse reactions are preferred, and those with significant toxicity are used only for severe unresponsive disease. The treatment ladder (Lebwohl, 2005) includes topical treatments (medicated ointments or creams, the first step), phototherapy (ultraviolet (UV) radiation, including oral psoralen and UVA, psoralen and ultraviolet A radiation (PUVA), the second step), and systemic treatment (including immunosuppressive drugs, methotrexate and cyclosporine, the third step).

An increased risk of cancer has been predicted among psoriasis patients because of the dysregulated immune function and the potential carcinogenicity of certain therapeutic regimens, such as UV exposure and immunosuppressant drugs (Arellano, 1997; IARC, 2000). An increased risk of skin cancer has been reported in most studies (Frentz and Olsen, 1999; Hannuksela-Svahn *et al*, 2000; Boffetta *et al*, 2001). However, for cancers in other sites, evidence is still limited because of the small numbers of patients involved and short follow-up times. We have carried out here a longitudinal cohort study to quantify the subsequent cancer risk in hospitalised psoriasis patients, using the Swedish nationwide registers; it is by far the largest to date, covering 15 858 patients and allowing a separate analysis of patients with multiple hospitalisations and thus with severe psoriasis.

## Patients and methods

We used the Swedish Hospital Discharge Register, founded in 1964–1965 by the National Board of Health and Welfare with a complete national wide coverage since 1987, to create a cohort of psoriasis patients. A total of 15 858 patients were retrieved from the registry according to the seventh (1964–1968, code 706), eighth (1969–1986, code 696), ninth (1987–1996 code 696) and tenth ICD code (1997, code L40). This cohort was linked to the national Swedish Cancer Registry, founded in 1958 with close to 100% coverage, to ascertain all incident cancers from the start of follow-up through 2004. The Cancer Registry used a four-digit code according to ICD-7 to identify tumours during the study period. Additional linkages were carried out to national census data to obtain individual occupational status, national Registry of Causes of Death to identify date of death,and the Emigration Registry to identify date of emigration. All linkages were carried out by the use of an individual national identification number that is assigned to each person in Sweden for their lifetime; it was replaced by a serial number for each person to provide anonymity.

Person-years were calculated from 1 year after the last hospitalisation for psoriasis until diagnosis of cancer, death, emigration or closing date (31 December 2004), whichever came first. The follow-up time was divided into three periods: 1–4, 5–9 and ⩾10 years. Standardised incidence ratios (SIRs) were calculated as the ratio of observed to expected numbers of cases. Expected numbers were calculated using the incidence rates for all individuals without a history of psoriasis, and the rates were standardised by 5-year age, gender, period (5 years group), socioeconomic status and residential area (Esteve *et al*, 1994). For cancers of the female reproductive system, rates were also standardized for age at first childbirth and parity. The 95% confidence interval of the SIR was calculated assuming a Poisson distribution, and they were rounded to the nearest two decimals (Esteve *et al*, 1994). All analyses were carried out using the SAS statistical package (version 9.1; SAS Institute, Cary, NC, USA). The ethics committee at Karolinska Institute, Stockholm, Sweden, approved this study.

## Results

Among the 15 858 patients hospitalised in Sweden for psoriasis during 1965–2004 9251 patients (58. 3%) were hospitalised only once, 2689 (16.9%) twice, 1255 (7.9%) three times, 1663 (16.8%) for more than three times. The follow-up time ranged from 0 to 40 years, with a median follow-up of 10 years. Only those cancers, which developed 1 year after last hospitalisation were calculated and a total of 1408 patients developed subsequent cancer after being hospitalised for psoriasis, giving an overall SIR of 1.33 (all 1+), as shown in Table 1. For specific cancer sites, only those cancer sites with at least 15 cases during the whole follow-up period were listed. The highest SIR of 2.97 was noted for oesophageal cancer. The risk for squamous cell skin cancer was significantly increased during the whole follow-up period, but for melanoma no increase was noted. The risks of cancers of the upper aerodigestive tract, stomach, liver, pancreas, lung, kidney, bladder, and nervous system and non-Hodgkin lymphoma were also significantly increased. The risk for some cancers, such as pancreatic cancer (5.63) and non-Hodgkin lymphoma (3.93), was very high during the (excluded) first year, probably because of a concomitant diagnosis. We further calculated the risks among male and female patients separately (data not shown). The risk of cancers in the upper aerodigestive tract, lung, kidney and bladder was somewhat higher among female patients, whereas the risk of live cancer was higher among male patients.

Multiple hospitalisations may reflect the disease severity, and we examined cancer risk among psoriasis patients by the number of hospitalisations (Table 2). The overall SIR was 1.25 for psoriasis patients who have been hospitalised once, and it was increased to 1.40 for those with 2–3 hospitalisations and to 1.61 for those with more than three hospitalisations. The SIR was higher for almost all the cancer sites when psoriasis patients had multiple hospitalisations, with an exception for upper aerodigestive tract and stomach cancers. We tested for the significance of the trend in the number of hospitalisations. Oesophageal and squamous cell skin cancers showed an increasing trend with the number of hospitalisations.

To study effects in relation to the changing therapeutic regimens and diagnosis criteria, we analysed cancer risk among psoriasis patients who were hospitalised in the 1970s, 1980s and 1990s (data not shown). The overall SIR was marginally higher among patients hospitalised in the 1990s compared with those hospitalised earlier. For specific cancer sites, the risk did not change during the study periods. We tested for the significance of the temporal trends, which was increased only for stomach cancer.

## Discussion

In this population-based study of 15 858 psoriasis patients identified from the Swedish Hospital Discharge Register, a 33% excess incidence of overall cancers was noted, largely attributable to cancers of the oesophagus, skin, upper aerodigestive tract, lung, stomach and liver and to non-Hodgkin lymphoma. This study has the advantage that it includes individual data on an entire population. The additional strengths include its prospective design and completeness of follow-up of psoriasis and cancer patients. All the data came from nation-wide databases guaranteeing reliable information. One limitation is that because of multiple comparisons some findings may be due to chance, while another is the lack of information on treatment and on possible confounding factors, such as alcohol drinking and tobacco smoking. Moreover, our data will not directly applicable to all patients with psoriasis as hospitalised patients must represent a severe subgroup.

The increase in overall cancer incidence is consistent with a few earlier studies from the Nordic countries (Frentz and Olsen, 1999; Hannuksela-Svahn *et al*, 2000), and also one from Sweden using patients hospitalised before 1983 (Boffetta *et al*, 2001), somewhat overlapping with our study population. Possible mechanisms for the increase of cancer risk could be immunological, that is, general tendency to cell proliferation beyond the epidermis (Boffetta *et al*, 2001) or carcinogenic effect of treatments, especially for the increase of squamous cell skin cancer and non-Hodgkin lymphoma, both of them are related to immunological deficiency. However, other factors, such as smoking and alcohol drinking are important. The highest risks were noted during the first year after last hospitalisation for psoriasis, which could be due to lead-time bias because of concomitant diagnosis.

For specific cancers, the increased risk of squamous cell skin cancer has been noted by many earlier studies (Chuang *et al*, 1992; Hannuksela *et al*, 1996; McKenna *et al*, 1996; Frentz and Olsen, 1999; Hannuksela-Svahn *et al*, 2000; Boffetta *et al*, 2001). The increase is thought to be due to UV exposure, but it may be increased by concomitant treatments. The risk for melanoma was close to unity. The risk for non-Hodgkin lymphoma was marginally increased, though confined to less than 5 years after hospitalisation. As this malignancy can be a sign of dysregulated immunity, it may be relevant to the evidence that severe psoriasis patients may receive immunosuppressive drugs. For nervous system tumours and leukaemia, the increase was confined to specific follow-up periods and chance cannot be excluded.

As alcohol drinking and tobacco smoking are often reported to be associated with psoriasis (Poikolainen *et al*, 1990; Naldi *et al*, 1992; Schafer, 2006; Favato, 2008), including one Swedish study (Lindegard, 1986), the increase in cancers of the upper aerodigestive tract, oesophagus, stomach, liver, pancreas, lung, kidney and bladder could reflect these factors. Unfortunately, information about individual drinking and smoking habits was lacking in this study, precluding an examination. The magnitude of alcohol and/or smoking effects on psoriasis has been evaluated in many earlier studies, but the data were heterogeneous because most of them were limited by small sample sizes (Schafer, 2006). In a recent population-based study involved 5687 hospitalised psoriasis patients in Finland (Poikolainen *et al*, 1999), where the health care system and diagnosed criteria were similar to Sweden, the mortality for alcohol and smoking-related causes was significantly increased among these patients compared with general population, with a standardised mortality ratio of 2.14 (men) and 1.47 (women) for alcohol-related causes and 1.44 (men) and 1.61 (women) for smoking-related causes, respectively. For these alcohol and smoking-related cancer sites, the risks were somewhat higher in female patients, with an exception of liver cancer, suggesting that stress could be related to the association between psoriasis and smoking and alcohol consumption.

We examined the data according to the number of hospital visits, using this as a surrogate for disease severity and chronicity. The overall cancer risk was associated with the number of hospitalisation. The excess in oesophagus, pancreas, lung and squamous cell skin cancers were most pronounced for patients with multiple hospitalisations. The highest risk of 6.97 was noted for oesophageal cancer among patients hospitalised more than three times. Multiple hospitalisations witness about the severity of the disease, but, in addition, the highly increased risk of oesophageal, skin and pancreatic cancers should be an extra concern, which may be indications for medical surveillance.

The diagnostic criteria and treatment for psoriasis patients have changed during the follow-up period, and we examined the temporal trends of cancer development among these patients. For most specific cancers, the risk did not change during the study periods. Topical corticosteroids are the most commonly prescribed treatments for psoriasis in the world (Lebwohl, 2003). Topical tars were ever used as an essential component of the Goeckerman regimen, but the use of tars has decreased greatly because of its staining on the skin. Phototherapy is a standard treatment for patients with moderate-to-severe psoriasis who have not responded to topical therapies (Lebwohl, 2003). Psoralen and ultraviolet A radiation (PUVA) was developed in the 1970s and has been used by some 13% of the Swedish psoriatics in the 1990s (Zachariae *et al*, 2001). Methotrexate has also been used for many years in the treatment of psoriasis, but its use was limited because of hepatotoxicity (Lebwohl, 2003). Cyclosporin alleviates psoriasis by inhibiting the immune system, which has been used for the treatment of psoriasis for more than a decade ago (Lebwohl, 2003). However, its long-term use is associated with nephrotoxicity and it has been limited to a maximum use of 1 year recently, thus its carcinogenetic effect could be largely excluded. In view of the various medications used in psoriasis treatment during the follow-up periods, the relatively constant risks observed throughout the study period suggest that, besides the therapeutic effect, psoriasis itself, with the contribution by smoking and alcohol drinking, may be associated with an increased cancer risk.

A 33% excess incidence of cancer was noted among psoriasis patients, involving squamous cell skin cancer and cancers related to alcohol drinking and tobacco smoking.

## Figures and Tables

**Table 1:** Standardised incidence ratios (SIRs) for subsequent cancer in patients with hospitalised psoriasis by follow-up time

	**Follow-up interval (years)**															
	**1–4**				**5–9**				**⩾10**				**All 1+**			
**Cancer site**	**O**	**SIR**	**95% CI**	**O**	**SIR**	**95% CI**	**O**	**SIR**	**95% CI**	**O**	**SIR**	**95% CI**				
Upper aerodigestive tract	15	**2.38**	1.33	3.93	12	1.91	0.98	3.35	21	**1.79**	1.11	2.74	48	**1.97**	1.46	2.62
Oesophagus	10	**4.30**	2.05	7.94	6	2.52	0.91	5.52	12	**2.54**	1.31	4.45	28	**2.97**	1.97	4.30
Stomach	16	1.62	0.92	2.64	10	1.07	0.51	1.98	26	**1.57**	1.02	2.30	52	**1.45**	1.08	1.91
Colon	22	1.11	0.69	1.68	27	1.34	0.88	1.95	33	0.83	0.57	1.16	82	1.03	0.82	1.27
Rectum	13	1.16	0.61	1.98	17	1.49	0.87	2.40	24	1.05	0.67	1.56	54	1.18	0.89	1.55
Liver	15	**1.94**	1.08	3.21	13	1.71	0.91	2.93	29	**2.08**	1.39	2.99	57	**1.95**	1.47	2.52
Pancreas	15	**2.00**	1.12	3.31	6	0.82	0.29	1.80	20	1.49	0.91	2.30	41	**1.45**	1.04	1.97
Lung	42	**1.99**	1.43	2.69	37	**1.73**	1.22	2.38	71	**1.71**	1.33	2.15	150	**1.78**	1.51	2.09
Breast	35	1.02	0.71	1.42	42	1.22	0.88	1.65	85	1.15	0.91	1.42	162	1.13	0.97	1.32
Cervix	4	1.11	0.29	2.87	4	1.15	0.30	2.97	11	1.63	0.81	2.93	19	1.38	0.83	2.15
Endometrium	12	1.56	0.80	2.73	10	1.30	0.62	2.41	8	0.53	0.23	1.05	30	0.98	0.66	1.41
Ovary	8	1.27	0.54	2.52	5	0.82	0.26	1.93	8	0.68	0.29	1.35	21	0.87	0.54	1.33
Prostate	44	1.12	0.82	1.51	38	0.92	0.65	1.27	96	1.03	0.84	1.26	178	1.03	0.88	1.19
Kidney	18	**2.27**	1.34	3.59	9	1.16	0.53	2.21	18	1.25	0.74	1.99	45	**1.50**	1.09	2.00
Urinary bladder	20	1.52	0.93	2.36	18	1.35	0.80	2.13	43	**1.58**	1.14	2.13	81	**1.51**	1.20	1.88
Melanoma	9	1.05	0.47	2.00	5	0.56	0.18	1.32	21	1.09	0.67	1.67	35	0.95	0.66	1.32
Skin, squamous cell	26	**2.56**	1.67	3.76	25	**2.35**	1.52	3.47	40	**1.74**	1.24	2.37	91	**2.08**	1.67	2.55
Nervous system	7	0.89	0.35	1.84	8	1.03	0.44	2.03	29	**1.90**	1.27	2.73	44	**1.42**	1.03	1.91
Endocrine glands	4	0.83	0.22	2.16	7	1.46	0.58	3.02	16	1.71	0.98	2.78	27	1.42	0.94	2.08
Non-Hodgkin lymphoma	27	**2.44**	1.61	3.55	9	0.79	0.36	1.51	24	1.03	0.66	1.54	60	**1.31**	1.00	1.69
Leukemia	9	1.91	0.86	3.63	10	**2.15**	1.02	3.96	8	0.89	0.38	1.77	27	1.47	0.97	2.14
All	401	**1.54**	1.39	1.70	333	**1.26**	1.13	1.41	674	**1.26**	1.17	1.36	1408	**1.33**	1.26	1.40

Bold type, 95% confidence interval (CI) does not include 1.00; underline type, 99% CI does not include 1.00.

**Table 2:** Standardised incidence ratios (SIRs) for subsequent cancer in psoriasis patients by number of hospitalisations

	**Number of hospitalisations**						
	**1**		**2-3**		**⩾4**		**Trend test**
**Cancer site**	**O**	**SIR**	**O**	**SIRs**	**O**	**SIRs**	***P*-value**
Upper aerodigestive tract	33	**2.18**	9	1.51	6	1.85	0.49
Oesophagus	6	1.03	13	**5.59**	9	**6.97**	**0.01**
Stomach	33	**1.48**	13	1.50	6	1.23	0.75
Colon	54	1.08	18	0.92	10	0.96	0.53
Rectum	35	1.23	12	1.08	7	1.18	0.88
Liver	33	**1.81**	17	**2.38**	7	1.81	0.72
Pancreas	21	1.19	7	1.02	13	**3.52**	0.06
Lung	92	**1.75**	37	**1.79**	21	**1.89**	0.78
Breast	104	1.10	41	1.23	17	1.13	0.66
Cervix	11	1.18	6	1.89	2	1.50	0.52
Endometrium	21	1.05	7	0.98	2	0.59	0.50
Ovary	11	0.69	6	1.07	4	1.59	0.12
Prostate	105	1.00	49	1.14	24	0.97	0.85
Kidney	28	1.48	10	1.38	7	1.81	0.76
Urinary bladder	51	**1.54**	16	1.21	14	**1.93**	0.74
Melanoma	24	1.00	8	0.92	3	0.73	0.62
Skin, squmous cell	34	1.26	29	**2.67**	28	**4.76**	**0.01**
Nervous system	29	1.44	8	1.10	7	2.01	0.68
Endocrine glands	16	1.30	6	1.35	5	2.31	0.31
Non-Hodgkin lymphoma	35	1.22	15	1.36	10	1.72	0.31
Leukaemia	15	1.29	9	2.04	3	1.32	0.60
All	835	**1.25**	358	**1.40**	215	**1.61**	**0.01**

Bold type, 95% confidence interval (CI) does not include 1.00; underline type, 99% CI does not include 1.00.
